# A Network Sensor Fusion Approach for a Behaviour-Based Smart Energy Environment for Co-Making Spaces

**DOI:** 10.3390/s20195507

**Published:** 2020-09-25

**Authors:** Teng-Wen Chang, Hsin-Yi Huang, Chung-Wen Hung, Sambit Datta, Terrance McMinn

**Affiliations:** 1 College of Design, National Yunlin University of Science and Technology, Douliou, Yunlin 64002, Taiwan; D10730018@yuntech.edu.tw; 2 Department of Electrical Engineering, National Yunlin University of Science and Technology, Douliou, Yunlin 64002, Taiwan; wenhung@yuntech.edu.tw; 3 School of Electrical Engineering, Computing and Mathematical Sciences, Curtin University, Bentley, WA 6102, Australia; Sambit.Datta@curtin.edu.au; 4 Shunya Pty Ltd., 44 Marcus Avenue, Booragoon, WA 6154, Australia; t.mcminn@shunya.com.au

**Keywords:** internet of things, solar energy, smart buildings, user behaviour, ambient agents, wireless sensor networks

## Abstract

User behaviour and choice is a significant parameter in the consumption patterns of energy in the built environment. This paper introduces a behavior-based approach for developing smart energy applications. With the rapid development of wireless sensor networks and the Internet of Things (IoT), human-computer interfaces can be created through the mapping of user experiences. These applications can provide users with dynamic feedback on their energy consumption patterns in their built environment. The paper describes a “Sensible Energy System” (SENS) that is based on user experience design methods with sensor network technology. Through SENS, solar energy simulation is combined with device consumption data in order to achieve an IoT network to facilitate the interaction between user behaviors and electricity consumption. The interaction between users and devices through SENS can not only optimize power consumption, but also provide consumers with additional choice and dynamic decision making regarding their consumption. This article provides an (1) understanding and analysis of users’ spatial interaction, explains the (2) planning of the new smart environment design and user experiences, discusses (3) designing a suitable Wireless sensor network (WSN) agent and energy connection, describes (4) the information that has been collected, and (5) incorporates a rooftop solar potential simulation for predicting energy outputs into the sensor network model.

## 1. Introduction

Through the introduction of technology coupled with software-based services, it has now become a reality to create smart environments that allow for users to interact more seamlessly with their immediate environment. Smart home/environment architecture focus on developing suitable cooperation between users and devices for the optimal utilisation and improvement of the quality of experience and service. Thus, the goal of the smart environment is for the user to easily control and regulate appliances with IoT-enabled sensors.

This paper focuses on the design of user engagement with energy consumption and interfaces to smart sensor networks within buildings. It describes the main technological components of user behavior, smart sensors, and smart energy connections in order to monitor and visualize energy use and generation within the built environment. The intention of IoT-enabled sensors was to motivate users to modify their use towards a comfortable and energy-efficient environment; however, traditional energy planning is a static design. Usage habits must be used to modify energy consumption to efficiently foster change.

End user experiences can play a crucial role in the uptake of smart sensor technologies. Within this context, this paper investigates a model to link end-users with energy sources within an off grid system. It draws from behaviour based design research to create an inventory of common motivators, principles, enablers, and barriers for behaviour change amongst end-users.

Thus, in this study, three different approaches were applied for the Sensible ENergy System (SENS) project issues [1] and experiment at co-making space. Virtual information can be converted to entity information and the interaction of users can become physical by using this architecture and a combination of the application of a digital interface.

### 1.1. Background

The theoretical framework for the development of a smart environment has been accelerated by the advent of flexible, configurable tools in contemporary smart home practices, such as spatial interactions for sensible space exploration [2,3,4,5] and mapping from physical to digital infrastructure sensors [6,7,8] proposed theoretical models for connecting virtual models to smart physical models [9,10,11,12].

The aim of the smart environment is to allow users and devices to respond to changes in their environmental context. Co-worker is a dynamic issue and, while using the static configuration, it can not increase the usage and the space interactive. Co-making space need the dynamic calculate method and source distribute. This approach was based on smart home design and developed the co-making space design. SENS design has been made feasible through advances in the integration of smart energy, smart environments, and user behaviors through innovative design methods and processes. Smart energy calculates models [13,14,15] and the development of hardware components for Internet of Things (IoT)-enabled sensors.

A smart city allows for people to seamlessly engage with their built environment [16]. The smart energy factor is used as evidence that smart sensors for comfortable and energy-efficient buildings and building management have created a new era for both sensing technology, sharing renewable and computational processing in order to facilitate the vision of smart environments [12,17]. Although challenges exist in their deployment, like residential buildings, would shift themselves toward modern households, use must be universally increased to further facilitate their use [18]. There are several approaches to increasing their usage, but user behaviors are key to refining interactions to change a smart environment and the IoT-enabled sensor design.

Design based on behaviors is an interactive activity that invokes different interactions among various knowledge entities with different design problems in dynamic design situations [19]. In human-computer interaction paradigms, such as mixed-initiative [20], dynamic interfaces [21], distributed interaction [19], and user experience [22,23] proposed theoretical frameworks for the conceptualization of physical to digital interface components and their interaction with complex environmental changes as well as internal and external stimuli.

All of the IoT-enabled sensors have their own agent technology that understands the user, and they can control various home appliances intelligently, therefore creating a more comfortable environment. The agent will be based on the single areas’ user to find out their own consumption demand and the agent will help the user in planning the energy and smart environment scheduling automatic and give the feedback to the user. At the same time, a smart sensor provides users with a real-time feedback mechanism for energy consumption, achieving the goal of energy-saving and emission reduction without influencing users’ daily normal usage [7]. Thus, a smart sensor can help users to simulate their behaviors and current usage to receive feedback and advice.

For the co-making space, the energy switch demands will be bigger than the general households. To work in the most energy-efficient manner while taking advantage of low energy tariffs, the user can take advantage of a reduction in expenses and energy use [24]. Electricity usage in a co-making space is relatively large, due to the use of large machines to meet their needs and the consumption will have higher amplitude. The smart grids [25], which are characterized by a two-way flow of both electricity and information, would effectively exploit the existing power grid to reduce or to eliminate blackouts, voltage sags, and overloads [18]. Therefore, the smart grid is a concept that integrates information and communication technologies (ICT) with grid power systems to achieve efficient and smart energy generation and consumption [26].

A single module is not sufficient for different types of behaviors and usage requirements, such as co-working space, co-making space, and smart environment. Smart energy shifts the energy design from static to dynamic, which can be used for smart energy design [27]. When the traditional configuration and hardware design change the user behaviors must need to re-investigate the demands and the context-aware integration can help the research to seek the user relevant times and the demand immediately. This definition of the context-awareness [28,29,30] it is mainly able to transmit the information that is required by the user to the place where the user can use according to different geographical environments, provide the appropriate information through the assistance of the sensor and according to the situation factors at that time, and realize it in a wireless network environment. Additionally, it allows the developers of services to define a relevant context for a given scenario, and the information can be used to describe a situation during an interaction with the system.

### 1.2. Motivation and Approach

The purpose of this study is to test a behavior-based smart energy environment with a focus on improving the energy efficiency of co-making spaces. The motivation for the project is to improve the usage rate of the facilities of the co-making space, to increase the number of interactive spaces for users, and to maximise the use of dynamic energy for power consumption.

Applications that are based on Wireless Sensor Networks (WSNs) must support reconfiguration with minimum human intervention depending on dynamic context changes in the environment [31]. Based on the spatial interactions and embedded information of a space, the smart environment has been used in many aspects with many technologies, such as sensor network smart environment and co-making space exploration. Each tool provides feedback to users, which is critical in determining the relationship between usage and the environment’s surroundings.

For this project, the hypothesis was that all of the users can have an agent system and that the agents can change the device’s effect via users’ behaviors. The user is not the static identity, it has the multiple usage. We cannot use the single identity to compute their behavior, it needs dynamic compute and analysis. Thus, users can understand not only their consumption and daily behaviors, but also their investment in the energy that is generated to improve the usage experience.

The SENS project was establish on the co-making space usage; it included smart energy, smart sensors, and user behaviors to offer a smart environment design for a new spatial interaction and identify the sensor layers’ transfers and connections.

## 2. SENS Framework

The aim of this project is to help users understand the energy patterns of their built environment and identify potential sources of energy savings based on user choice and behaviour modification. While a WSN can record user activities, behavior trajectories, and usage, it cannot change usage habits. The SENS environment proposes new avenues for design and interaction for smart environment system design and planning of dynamic interaction behaviors. With this in mind, the following design criteria are proposed for the development of SENS:

**Agent Communication framework.** Role-play theory is applied to model the interactive behaviors of agents. The daily usage of each user was found to be different through observations and recordings.

**Smart Home Environment.** Smart energy should be decomposable into an aggregation of embedded consumption/generation information of a space. The embedded information minimizes complexity and provides a coherent integration between interactions and the environment;

**Smart Energy Environment.** This discrete agent should support open-ended, reprogrammable sensor input-output with the environment. Components should be reprogrammable and re-configurable to allow for a broader range of experiments with interactivity. The smart sensor consumption device connects using a Wireless sensor network (WSN), and the generation of energy connects through a Global System for Mobile Communications (GSM) and it is calculated by the Global Max Power Point (GMPP) tracking model. The GMPP method can improve the stored effect and the results of the PV. The smart environment includes a customized integrated energy system grid combining peripheral structure or cladding elements.

**User Interaction.** User interaction is a key criteria for SENS and aims to create a platform for behaviour-based smart energy through a user-centered methodology involving iterative prototype design to refine the key factors in assimilating users into dynamic sensor networks.

### 2.1. Agent Communication Framework

Simulating and predicting behavior can be done by adopting the Acting Role Model (ARM) [32], agent technologies, and intelligent dynamic interactions with the design system, called the Dynamic Agent-Based Role Interplay System (DARIS), in order to examine interactive behaviors. The ARM system has five main components: roles, actors, stages, scripts, and scenes. Each component has a task with other components based on definitions and mechanisms for interactions. Thus, the DARIS is based on the ARM system with three layers (internal design situation layer, inter-process layer, and external design situation layer) and five different types of agent entities (user agents, role agents, director agents, scene agents, and stage agents) [19].

**Figure 1:**
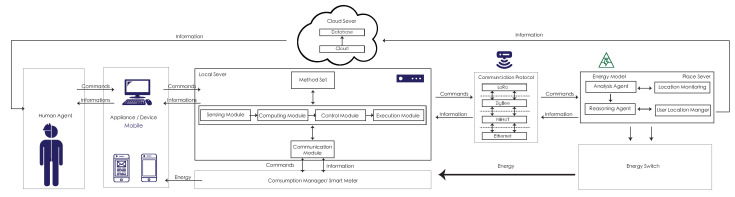
The system structure of the Sensible Energy System (SENS) project.

Therefore, the structure of the system in Figure 1 is divided into the physical and the virtual environments. On the left side of the figure is the real-world design, which configures the physical device and user behaviors. Thus, in the physical design, when the user interacts with the environment, the smart sensor begins tracking behaviors and uploads data to the local/place server. The local/place server data are uploaded to cloud services in real-time. The virtual environment design is on the right side of the figure, and the usage generated/consumed and user behaviors are converted to numerical and computational data in cloud services. In the interactive aspect of the process, physical data are recorded via the smart sensor to sense usage and behaviors to control the virtual environment and connect to changes in the physical object.

**Figure 2:**
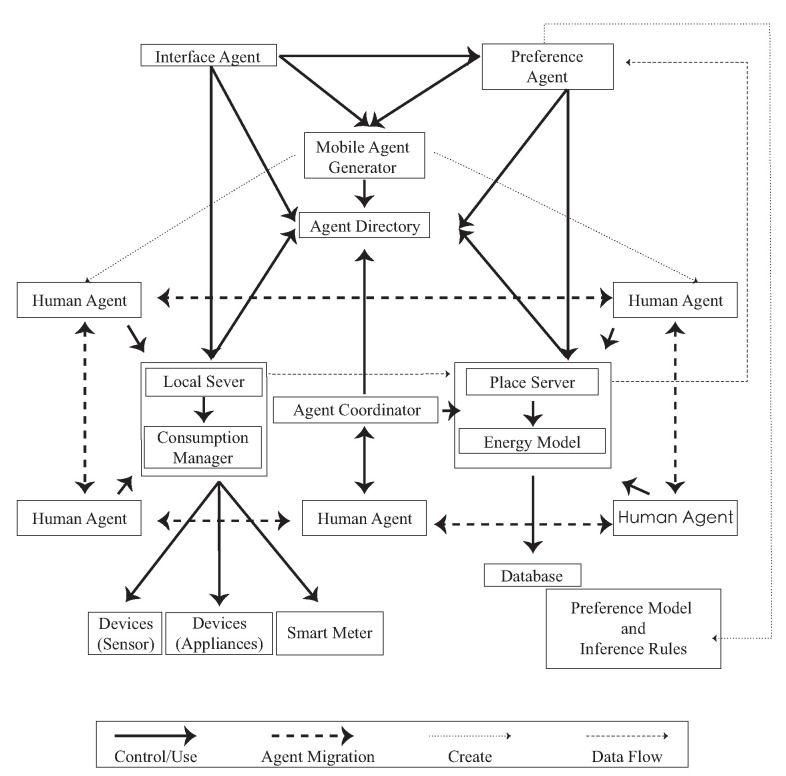
Smart sensor component data flow.

We used the smart sensor to be the sensing port to create a sensible energy space; in the environment, every physical object has its own ambient agent. The ambient agents in Figure 2 are divided into human agent, interface agent, preference agent, and sensible agent. When the user enters a space, the local server begins to track user behaviors and device consumption. The consumption data and user behavior are transmitted to the place server for integration, calculation, and analysis, and they are stored in the database.

The agent communication framework includes the human agent, an appliance, a local server, a consumption manager, a communication protocol, a place server, an energy switch, and a cloud server. The user is the human agent and it uses an appliance in the physical space. When the local server receives the user’s information, it begins interacting with other agents in the system environment via the sensing module, execution module, and communication module. The local server has a computational model that is responsible for processing and storing the perceived and received messages and a control module using the method and the information processed by the received computing module and the information to communicate with other agents for further analysis and reasoning to make decisions for the communication and execution modules. All of the local server’s data are transmitted to the place server through the communication protocol. The place server includes the agent, reasoning agent, location monitoring, and user location manager. After transferring all of the information to the cloud server, the cloud server records the data and converts it to the human agent to show up and inform the user.

### 2.2. Smart Home/Environment Architecture

Along with the development of wireless communication, a sensor module containing a sensor unit and a wireless transmission unit can transmit the sensed information to the information center wirelessly. Such sensing modules can be distributed in different locations to form a WSNs and transmit the sensed information back to the control center. Ubiquitous smart environments that are equipped with low-cost and easily deployable WSNs and widespread mobile ad hoc networks (MANETs) are creating new opportunities for wide-scale urban monitoring. The convergence of MANET and WSNs paves the way for the development of new IoT communication platforms with a high potential for a wide range of applications in different domains [33].

A multi-device mapping approach to components and connections is adopted. The architecture that was developed for smart environment and its subsequent articulation with components is adopted. The smart environment architecture is rationalized in order to account for both part-whole relationships of the IoT components as well as the dynamic generation/consumption of energy.

The smart environment design includes ZigBee to combine the agent system. ZigBee has some benefits, such as low power consumption, low cost, large mesh capacity, small effective range, and a flexible working frequency band, which is why this WSN was used. In the agent design, the user’s behavior signals are input into the agent system to determine the action, and its transfer type is the “string” to the corresponding device. Thus, Zigbee is an effective WSN delivery medium with a transfer speed between 10–250 kb/s, and it will not exceed 20 bytes. This characteristic is very suitable for sensing in a smart environment.

### 2.3. Smart Energy Environment

The system design proposed in this paper aims to help users to identify energy consumption behavior patterns and understand the energy patterns of their built environments and identify potential sources of energy saving. The energy information could normally be missed in the transfer process, but the missed information can enable the user to make energy consumption decisions and generation decisions.

The energy information could normally be missed in transfer process, but the missed information might enable the user to make energy consumption decisions and generation decisions. The co-making space is flexible and complex, and it has different user demand, space types, usage type, and multi-factors that are interconnected to each other. Therefore, we need to find out the experiment space demand, such as space configuration, user behavior, machine usage status, usage frequency, and the renewable generation and the detail will introduce in this section.

### 2.4. User Interaction

The SENS process integrates real-world and virtual environments based on a smart home environment/architecture concept, which involves a system in which physical entities and virtual information are referenced to each other in a recursive manner through a series of physical changes, information analysis, and user behavior suggestions. In order to integrate virtual and physical environments, smart sensors and smart energy must be combined to allow the user to observe usage and behaviors in the physical space and interact with the virtual object to improve device usage.

In this paper, a suitable wireless structure has been discussed, and encouraging user behaviors to be in compliance with the results and the combination of the solar PV and the smart grid is the next step. The GPMT has an excellent transmission effect and it is more effective in storing and tracking energy. After that, the energy information is sent by the GSM to the cloud server. The system will through the user’s behavior and usage to iteration the feedback of consumption suggest. Transparent information is the most important feature for the average user, because most designs cannot easily show the causality and need to switch devices in order to compare and understand the data.

Such sensing modules can be distributed in different locations and connect the green energy, consumption energy, and user behavior to form WSNs in order to transmit the sensed information to the control center analysis and represent. The analysis data usage rate can increase the system, which maximizes the shared space and decreases the daily usage. The users only need to operate the interface agent that receives the calculated data to change their behaviors.

The next sections describe the development of the SENS design prototype based on user experience design methods with sensor network technology within a physical co-making case-study, the Idea Factory at NYUST in Taiwan.

## 3. Co-Making Spaces: Idea Factory

Co-making is an arrangement in which users can share office space, allowing for cost savings and convenience through the use of common infrastructure, such as equipment, utilities, and receptionist and custodial services and, in some cases, refreshments and parcel acceptance services. Additionally, co-working helps workers to avoid the isolation they may experience while telecommuting, traveling, or working at home, while also eliminating distractions.

The Idea Factory is a co-making space providing shared large and high power consumption machines for their work. Idea Factory is a platform for people-oriented creators from either the engineering or the design domain to learn, think, and make by providing interdisciplinary workshops in both physical and virtual spaces. An experimental system was used to evaluate and identify the potential interactions that are desirable to users to identify a contextual scenario that is suitable for this research.

The Idea Factory contains several distributed thinking corners that allow makers to relax and to think. All of the innovators of the learning space and the flip classroom are connected either virtually or physically in order to provide dynamic and feasible learning spaces for makers.

**Figure 3:**
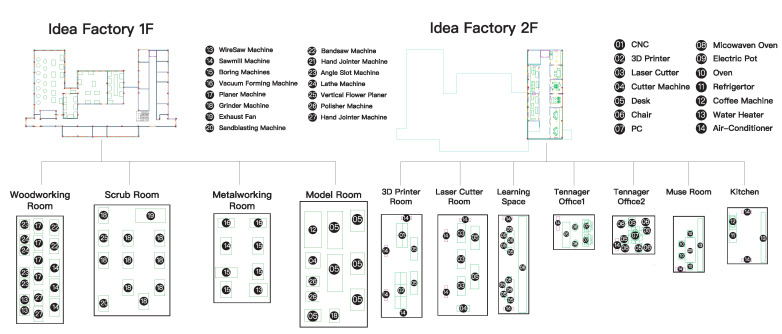
The facilities of the Idea Factory.

The facilities (Figure 3) are similar to a smart factory. In this factory, we have the co-worker, co-maker, normal office staff, and some part-time participants. For making, the facilitation and management of design, digital, and craft workshops are integrated together to offer a seamless working pipeline. In this implementation space, it has professional equipment, such as carpentry, metalworking, a laser cutter, a 3D printer, CNC, and robotic arms and workshops. This space has the multiple persona, but we will focus on the co-making and co-making user’s behavior. We believe that every behavior can be described, and they not only use the energy, but also generate the energy.

### 3.1. Agent Design

**Figure 4:**
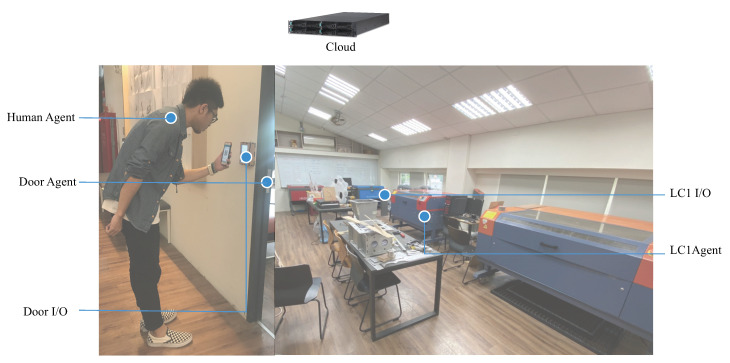
Agent communication in the co-making space.

In addition to the sensor network, agents are designed for retrieving the behavioral data in terms of coordinating the energy consumption with machines usage in the co-making environment following the communication framework that is shown in Figure 2. The following example demonstrates one interaction sequence on the use of the laser cutter device that involves three major agents: human, LC1 (the laser cutter machine 1, the local server), and 207 (the laser cutter room, the place server) with two I/O: Door I/O and LC1 I/O. The agents are designed to coordinate and adapt user behaviour, not decrease energy consumption directly, as shown in Figure 4.

**Figure 5:**
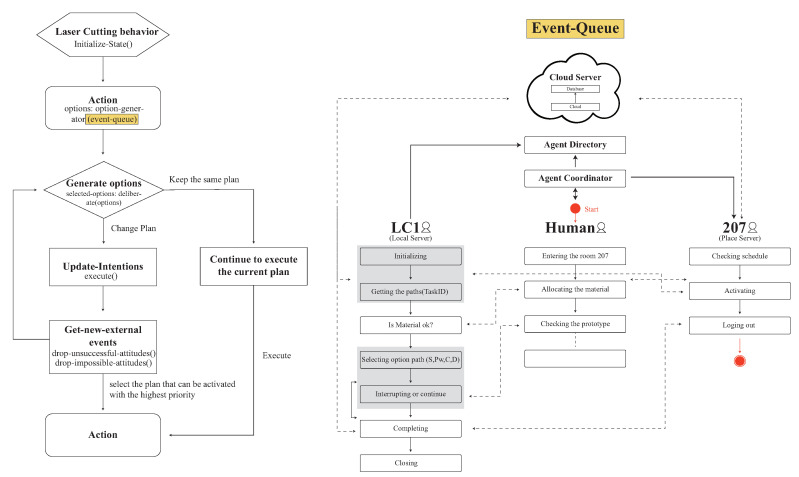
BDI model covering SENS agent framework.

Each agent represents a modular individual reasoning architecture in Figure 5. The advantages of that representation are in the ease of setting up, modifying, and expanding operational reasoning or tactics for various interaction scenarios within the building. Before users (Human Agent) enter the space, they will need to make a reservation on the machine that they are going to use in a time slot with proper dataset. In the laser cutting scenario, a set of paths with option on power and speed for each path need to be remotely input. The event sequence for the communication between the three agents are:

1. when user enters a space, the human agent informs agent coordinator, which in turn signals the 207 agent. The 207 agent will then check the user ID by communicating with cloud server;
2. if the reservation is valid, 207 agent will inform the door I/O to unlock the door and the LC1 agent associated with the reserved machine LC1 will be notified by agent directory;
3. once LC1 is activated, cloud server provides the paths with initial (speed, power) for each path by sending the task ID to cloud;
4. the sequential steps for LC1 with internal belief on paths are checking material (by communicating with human agent), then the loop over the path queue to record the energy consumption and duration for each path. A set of (path, speed, power, consumption), duration) data are recorded and sent back to cloud when the loop is completed;
5. if the interrupt signal is sent by the human, 207 (room) agent will be notified to provide comments either to continue or stop the process by checking the outcome of product;
6. when the process is completed, the data is sent back to cloud server. LC1 I/O is informed for shutdown sequence and 207 agent is notified for the total energy consumption for the task; and,
7. human agent interacts with 207 agent for logging out sequence and leaving the space.

The optimization and the normal option for the laser cutter path recorded can provide the energy consumption patterns, as well as possible user awareness on the energy consumption patterns; such an option will also affect the consumption result if the time is longer, the energy consumed is higher, but the laser result will be more detailed, as desired by making. Hence, every agent will communicate with each other and have their own task and goal and give the user the option for the optimization. The optimized options sometime might not produce the best output results, but will have lower consumption.

### 3.2. Smart Environment

The computing concept, especially ubiquitous computing, is often used to develop social applications for spatial interaction, spaces containing countless sensors, all static objects, and space itself as parts of a seamless Human-Computer Interface between habitats and their surroundings. This interface, or the so-called sensible environment, can sense habitants, conditions, behaviors, environmental facts, and contextual information. Thus, if we incorporate an alternate scenario, such as co-making spaces into the design, we will have the same experience of this kind of problem. When the context is a situation that can be used to describe characteristics of information, such information is considered by users and applied in a mutual interaction. Furthermore, computing records every motion, habitat, and behavior, such as working, cooking, and prototyping, according to the timeline, sets previous tasks as the target, and analyzes the activities in the spaces.

Some smart environment designers have not considered the interaction of users’ experiments with the environment. Therefore, SENS is a new experience design that has been implemented via smart environment equipment. The construction of general smart environment has a corresponding relationship with users’ habits, but the information does not correspond to each user’s interaction. Through the connection of objects in the IoT architecture, devices can transfer information to each other, increase user interaction with the environment, present information on a digital interface, and provide all of information in the most understandable way for the user.

**Figure 6:**
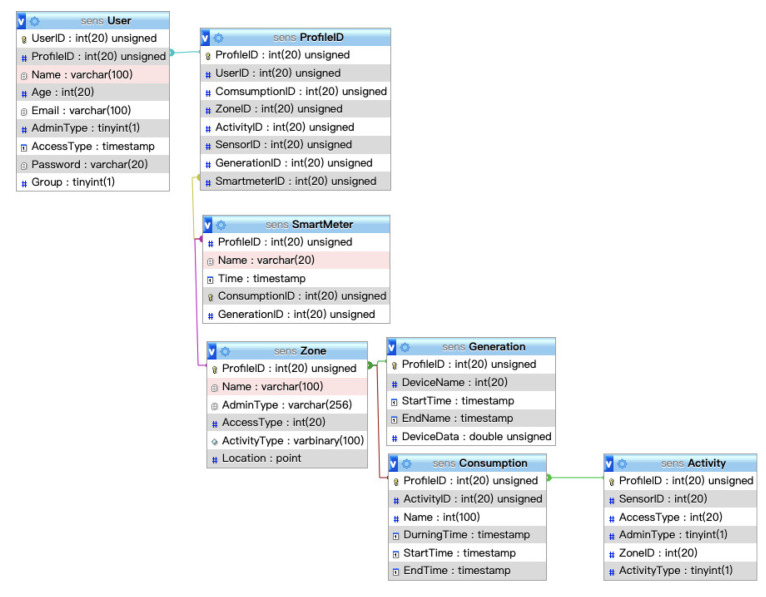
SENS brevity/readability UML diagram.

The brevity/readability UML (Figure 6) diagram shows how the smart environment architecture integrates the generation of dynamic energy with the sensors in the environment. When a user enters a space, the local server begins to track user behaviors and monitor device consumption. Sensors track user activity and monitor their consumption of energy within the Idea Factory. The consumption data and user behavior data are transmitted to the place server for integration, calculation, and analysis, and they are stored in the database. Users only need to operate the interface agent that receives the calculated data to change their behaviors. Along with the development of wireless communication, a sensor module containing a sensor unit and a wireless transmission unit can wirelessly transmit the sensed information to the information center.

### 3.3. Smart Energy from Rooftop PV

**Figure 7:**
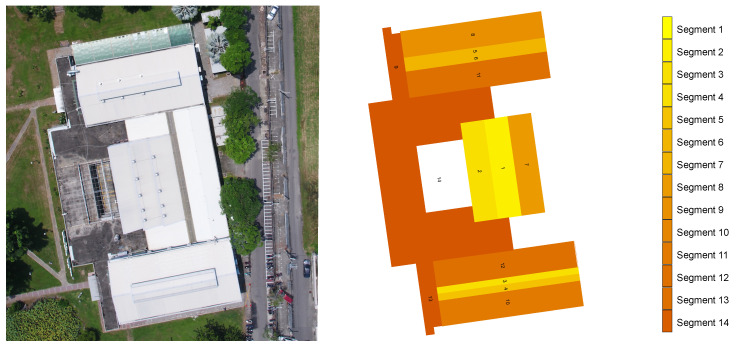
Plan View of Idea Factory Roof (**left**), three-dimensional (3D) model of the Roof showing roof segments for solar analysis (**right**).

Our exposition for smart energy thus far includes dynamic behaviour of consumption patterns, as described above. To calculate the generation of energy from a solar PV system, the rooftop of the Idea Factory is divided into 14 segments of the hardware system’s distribution in order to calculate it (Figure 7). The building location and roof geometry are used to simulate the solar energy potential to power the Idea Factory. The building model is geo-located while using a latitude/longitude pair or Open Location Code https://github.com/google/open-location-code/blob/master/docs/olc_definition.adoc. The latitude/longitude information can be inputted directly or via the Open Location Code (see google pluscode https://plus.codes/). Using long codes (up to 12 characters long) allows for resolution down to approximately 3.4 by 2.7 m area and they are readily convertible to latitude/longitude.

The roof geometry of the Idea Factory building comprises fourteen roof segments. A roof segment is defined as an ‘n sided simple polygon’ defining a single polygon of the roof. The polygonal chain does not intersect itself, although, in this case, the polygon can contain holes. Each roof plane has a single orientation, slope, and pitch. From the polygonal chain, the segment area is calculated and the panels are laid out for a horizontal and a vertical orientation.

In order to predict the amount of solar radiation at a specific point above a reflecting plane, the roof model is rendered using the Radiance Lighting Simulation Suite (referred to as Radiance in this document and available via http://www.radiance-online.org/). Radiance is an open source software that is capable of predicting physical accurate lighting levels on any plan [34]. Predictions using the Radiance Lighting Simulation Suite have been validated by a number of authors [35,36,37,38,39].

The technique used to generate the total solar radiation potential on the roof segments utilises a modified three-phase method, as described in Brembilla et al. [40]. The methodology creates a sensor point at the centroid of the roof segment. The interior matrix is not used in this application. The weather file (see http://climate.onebuilding.org/) that is associated with the project’s longitude/latitude is used to create 2305 sky patches based on the Perez [41,42] all weather model and hourly intervals.

Table 1 shows Average Hourly Solar Potential per roof segment (W). For the case of Yunlin county, Taiwan, a daily average of 3.52 could be generated, and 1284 kW of the relative power could be produced in a year. From the hourly (kW/$m^{2}$) simulated values, the monthly totals are computed as kW/$m^{2}$ for each roof segment. Additionally the area of the maximum number of panels either vertical or horizontal are multiplied by the simulated monthly totals to compute the potential roof segments solar potential. To compute the average daily and monthly contributions, the number of daylit hours for each month are compiled from the weather file and then used to compute the monthly hourly averages.

Each segment can generate different energy values for different months, hours, and days, and the choice of the hardware can increase the generation potential based on panel efficiencies. In this experiment, the Idea Factory had a latitude of 23.691 and a longitude of 120.533, and the data were collected via the central weather bureau and were calculated. Thus, all of the hardware system requirements could be determined. A 1.1 m by 1.7 m panel was used to generate energy, a system energy efficiency rating of 0.27, and temperature coefficient Pmax (air temperature variation about 25 °C), as shown in Table 2.

### 3.4. User Interaction

SENS is an integration of both real (devices) and virtual worlds (energy simulation) to create new environments and visualizations of physical and digital objects that co-exist and interact in real-time. This section presents the SENS interaction model while using a design-oriented user experience approach comprising reciprocal spatial interactions that operate based on a computation protocol and user behaviors.

**Figure 8:**
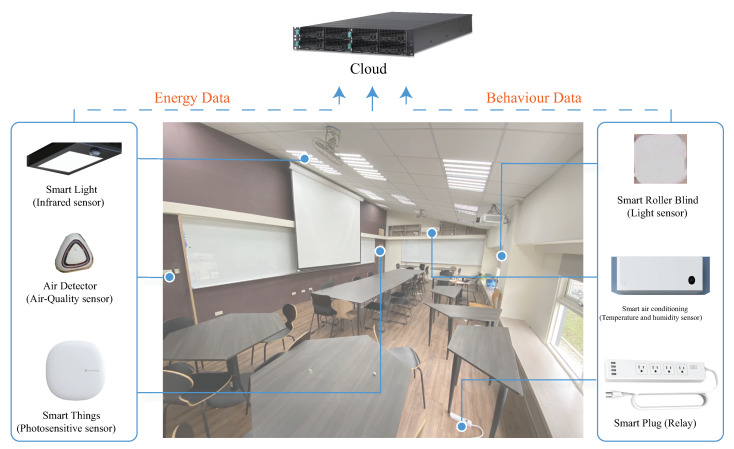
A hybrid Internet of Things (IoT) model of behavior/Energy data layers.

Figure 8 shows that the hybrid IoT model was constructed by decentralizing the smart sensors in the co-working/co-making environment. In our co-working implementation environment, the sensor choice included used an infrared sensor, ultrasonic sensor, air-quality sensor, photosensitive sensor, light sensor, temperature sensor, and humidity sensor. The basic smart sensor wireless transfer features, Arduino duemilanove, was used to connect the XBee to carry out ZigBee communication mode and achieve the energy control, switch, and transfer to consumption manager. Additionally, in the co-working environment, as shown in Figure 8, each agent is implemented using Raspberry PI with ZigBee communication mode and achieve the energy control, switch, and transfer back to consumption manager as well.

The behavior data were based on the position of the customized sensor to record and then transfer the data to the local server and compute the behaviors and provide user feedback to the sensor. The main reason for using Xbee is because it can be used in various applications, from ZigBee to high-transmission, low-latency applications. Additionally, the host interface API is another important part of XBee. It is interchangeable and can handle different types of communications, including ZigBee, 802.15.4, and WiFi. Thus, all pf the smart sensors can record and transfer energy/behavior data every 15 min wirelessly to the cloud server in order to compute, analyze, and store the data. The cloud servers store all data and release the data during a suitable opportunity to translate them into the available data to display on the SENS interface to notify users.

**Figure 9:**
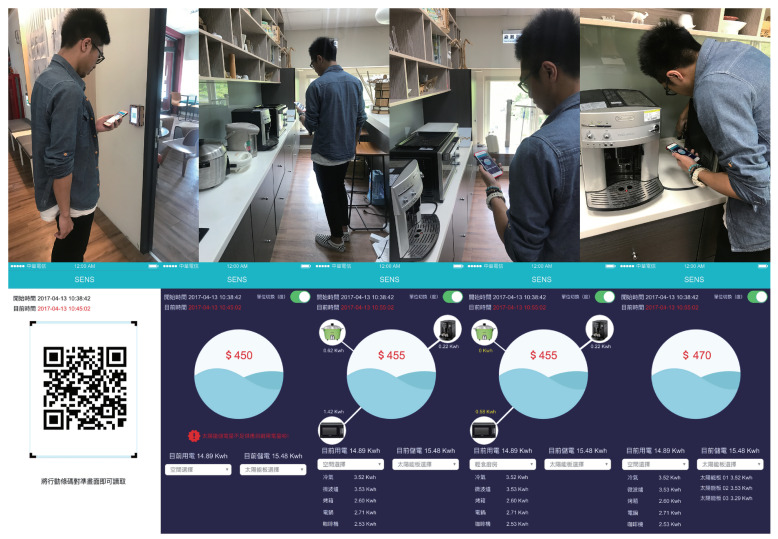
Scenario Simulation For the App and User.

In the interaction scenario (Figure 9), users enter each space of the Idea Factory with an app via the QR code access the screen on the door. After scanning the QR code, the energy consumption/generation of the location was displayed in the SENS with basic location information, such as room number and function.

When the user approaches the electronic devices that are located in the room, there are two ways to see the current consumption load of the device. First, through SENS, it is displayed as energy consumption information on the user’s mobile device. Second, the demand and use are displayed on a physical LCD with a notification sent to the user. Through the app, users can choose to evaluate how much energy they could save while the physical LCD tells the user what device are accessible in the zone. In the scenario if users evaluate the device usage and select the energy generation, SENS displays the amount of energy usage, source of energy, and the predicted monthly savings in dollar amounts if renewable energy (rooftop solar simulation) is utilised.

Summarising, the SENS prototype combines smart sensing, sensor network design with user experiences in order to develop and test a behaviour-based smart energy environment. The prototype includes both the consumption and the generation of energy within shared co-making spaces. In the co-making spaces, each user has a different usage profile, behaviors, and energy usage that affect the daily environment and energy consumption.

## 4. Evaluation of User Behavior in SENS

The SENS environment that is described in the previous sections provides new avenues for design and interaction for smart environment system design and planning of dynamic interaction behaviors. All devices and their efficiencies must be evaluated with respect to users in order to obtain an effective understanding of the co-existence and interactions in real-time design.

An evaluation study user behaviour with SENS was conducted daily over a month (July 2019). Six users were provided with an Idea Factory smart app application (Figure 9) on their mobile phones that was connected to the sensor network and energy prediction modules. Their access and egress to the building, internal movement within the Idea Factory, device usage patterns, and energy consumption were tracked using the SENS implementation.

This section presents the evaluation of users’ activity and how they can interact with an environment of connected objects via IoT. The modular SENS design helps users to receive dynamic information on the energy consumption of their devices, as well as the predicted energy generation that is based on the rooftop solar PV simulation model described in Section 3.3.

The energy generation output was provided through the solar potential simulation engine with hourly average outputs. The sensor data and the simulation data were both combined into a cloud server, as shown in Figure 9.

**Figure 10:**
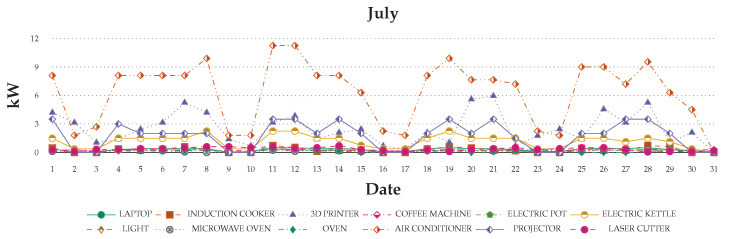
Monthly of every device usage.

The device’s recorded daily usage can show differences from the original configuration. Device operation, the idle mode, and the shutdown of energy consumption have different effects. Through the data record, the device situation can be predicted beforehand in order to change the device. Thus, all device consumption was recorded for one month to understand and simulate user scenarios (Figure 10).

### 4.1. Single Month Analysis

The interactions between users and the IoT connected devices can modify the behaviour of users in order to reduce the power consumption at the location. Users can also connect to view energy usage to access power generation information, such as the status of solar panel storage, through this device. Examples of use are shown in Table 3. In this figure, six different types of users’ weekly usage in the same space are presented. User *F*’s usage is the highest among all users, and have used most of the high consumption machines.

**Figure 11:**
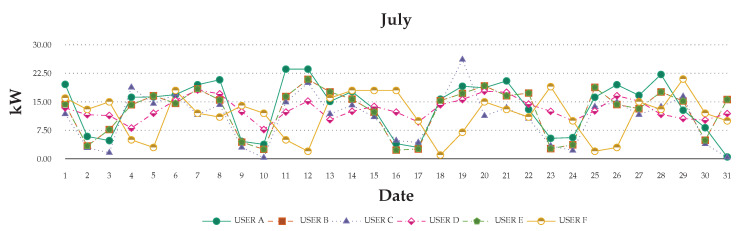
Consumption patterns of the daily usage of the six evaluated users over the month of July.

**Figure 12:**
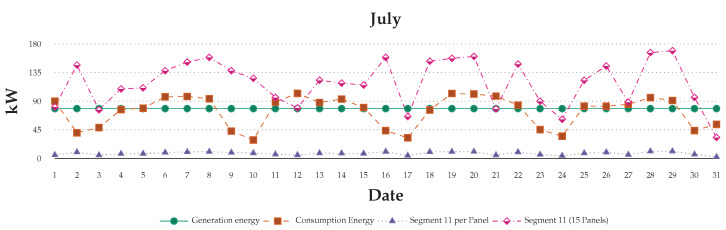
User data with simulated generation energy and grid backup power.

Figure 11 and Figure 12 show the recorded data for one month on a graph. The graph shows that the usage still has a different effect, even if a user stays in the same place each day. The usage will through system of the users’ requirement to change their behaviors and daily activities. Thus, the monthly record can help in understanding users’ basic behaviors and usage. The value increases and decreases is the way to explore daily in order to determine the research of the differences.

### 4.2. Single Day Analysis

**Figure 13:**
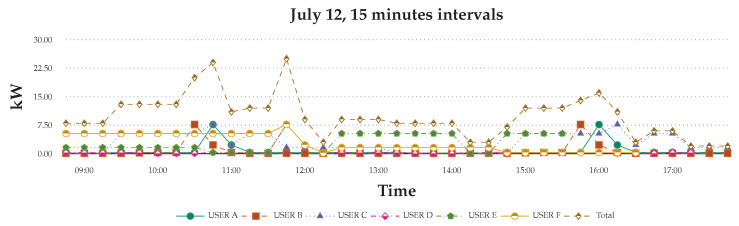
12 July Scenario: Comparison of 15 min consumption energy of the six users.

Figure 13 shows the recorded data for data on the graph. The graph shows that, even if the users stay in place a day, the safe still has different effects. The usage will through their behaviors and the activity to change. Single-day analysis can understand the difference between the users in every 15 min.

**Figure 14:**
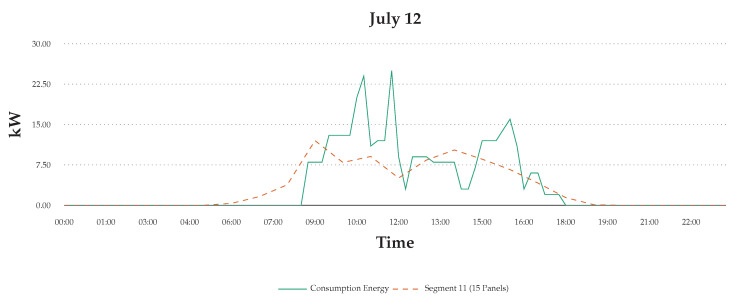
12 July scenario: consumption energy data with simulated generation energy at 15 min intervals.

Figure 14 shows that the consumption data that are shown in the blue line vary during the day and month-to-month, depending on usage and user behaviours. The green line in the figure shows the simulated energy generation data. The results highlight the degree of daily variance based on using 15 panel PV system on roof segment 11. However, this design simulates for the six users in the scenario described in the paper. However, the introduction of distributed storage daily fluctuations, such as the one observed in our test study, can be balanced with consumption patterns through prediction and simulation. As we observe in our experiment, human behaviors are complex and multiple factors determine usage patterns. There is no effective way to specify or predict energy usage increases and decreases during the day. However, the simulation feedback indicates that the visibility of consumption data in real-time can be an effective method in influencing change in their usage habits.

## 5. Conclusions

The goal of this research is to help users understand real-time energy consumption patterns within their built environment through the use of a sophisticated combination of sensing technologies, energy within a smart, connected, and aware environment. The purpose of this study is to test a behavior-based smart energy environment with a focus on improving the energy efficiency of buildings. The motivation for the project is to improve the usage rate of high power consumption facilities, such as co-making spaces, in order to increase the number of interactive spaces for users and maximise the use of dynamic and renewable energy, such as rooftop PV, for power consumption.

The paper presents the SENS (Sensible Space) framework, a platform for developing behaviour-based smart energy building environments through a user-centered methodology, in order to assist users in identifying potential sources of energy savings based on time of use and behaviour modification. Using iterative prototype design methods, the interaction sequences of users from entering the space, identifying making activities, and exploring energy consumption are recorded and analyzed. A novel agent based virtual twinning framework based on the user’s journey map, classes of behaviors, and devices and their interactions with energy and the environment have been proposed.

The paper presents the design and validation of the components of the framework in order to evaluate and understand the scenarios of end-users in an energy consumption situation. A use-case of a co-making space, the IdeaFactory, which combines the SENS components with the physical elements of energy usage, sensors, networks and user behaviors within a smart building environment are described. Based on the spatial interactions and embedded information in a space, each device provides energy consumption feedback to users, which is critical in determining the relationship between energy usage and the environment’s surroundings.

**Agent Communication.** Because the building activities are dynamic, agent-based communication is used to model multiple roles. The agent communication layer records user and device activities, behavior trajectories, and usage within the building. Through the incorporation and planning of dynamic interaction behaviors, the SENS environment proposes new avenues for the development of smart buildings.

**Smart Energy Environment.** Smart energy environments should be decomposable into a consumption/generation modules. In this project , the roof space of the building is utlised for driving renewable energy into the SENS framework. The smart sensor generation utilises a virtual simulation model that is connected to the Wireless sensor network (WSN) that tracks device consumption, and it is presented in real-time as a source of smart energy.

**User Interaction.** User interaction is the central criteria underpinning the development of a platform for behaviour-based smart energy. The user-centered software app and methodology are tested involving six users over a month. The data collected are analysed and presented in this paper in order to refine the key factors in assimilating users into dynamic sensor networks. The participants stated that, by making the energy information transparent and visual, they were better able to understand usage and change their behaviors.

In future work, time-of-use (TOU) pricing based on interactions between energy peak time, half-peak time, and off-peak time will be further explored in order to map energy consumption. Another area of work is the use of distributed energy storage in order to extend the useful hours of operation beyond solar PV generation daylight hours. A third area of investigation is to impose peer-to-peer interactions in terms of energy credits stored within the SENS framework. In summary, the record of user experience can be used to not only to identify energy demand for co-making infrastructure, but also modify the the usage behaviour. Through iterative design, constant corrections and fine-tuning energy consumption patterns in buildings can be positively impacted in order to ensure users and device usage are informed. Furthermore, the introduction of distributed energy generation, such as through rooftop solar, can serve to modify user behaviour and integrate everyday energy usage with sustainability goals.

## Figures and Tables

**Table 1:** Average Hourly Solar Potential per roof segment (kW).

	Jan	Feb	Mar	Apr	May	Jun	Jul	Aug	Sep	Oct	Nov	Dec
Day Lit Hours	367	336	372	404	434	420	**434**	434	390	378	331	341
1	0.902	1.024	1.272	1.341	1.307	1.589	**1.734**	1.775	1.575	1.303	1.069	0.991
2	0.915	1.029	1.262	1.348	1.307	1.563	**1.699**	1.779	1.585	1.313	1.07	0.971
3	0.334	0.396	0.488	0.523	0.527	0.62	**0.675**	0.69	0.597	0.499	0.395	0.355
4	0.439	0.474	0.567	0.562	0.532	0.61	**0.666**	0.731	0.692	0.617	0.516	0.477
5	0.325	0.393	0.491	0.529	0.529	0.627	**0.686**	0.696	0.605	0.499	0.398	0.348
6	0.439	0.474	0.568	0.562	0.532	0.61	**0.667**	0.732	0.693	0.617	0.516	0.476
7	0.977	1.111	1.391	1.449	1.399	1.687	**1.841**	1.928	1.688	1.444	1.18	1.06
8	1.172	1.505	1.882	2.043	2.059	2.467	**2.666**	2.713	2.342	1.883	1.497	1.266
9												
10	1.781	1.861	2.274	2.203	2.051	2.373	**2.634**	2.831	2.725	2.448	2.058	1.937
11	1.762	1.879	2.276	2.209	2.088	2.38	**2.638**	2.887	2.773	2.449	2.084	1.907
12	1.17	1.495	1.864	2.023	2.053	2.436	**2.678**	2.71	2.335	1.887	1.481	1.273
13												
14	7.62	8.717	10.699	10.782	10.517	12.34	**13.587**	14.105	13.01	11.076	9.17	8.24

**Table 2:** Roof Segment layout showing the roof parameters and the maximum number of vertical and horizontal panels.

Roof Segment	Area	Orientation	Pitch	Max Number of Panels	
	m ${}^{2}$			Horizontal Panels	Vertical Panels
1	311.492	82	25	119	100
2	311.492	82	25	119	100
3	124.2	352	13	25	36	
4	124.024	172	13	25	36
5	123.789	352	13	25	36
6	123.615	172	13	25	36
7	311.493	82	25	119	100
8	467.431	352	16	175	144
9	0	346	16	-	-
10	450.098	172	15	175	144
11	467.116	172	16	175	144
12	467.432	352	16	175	144
13	0	258	76	-	-
14	1908.389	90	0	729	729

**Table 3:** Usage in the experiment space.

		User A	User B	User C	User D	User E	User F	User G	User H	User I
Laser Cutter Room	Laser Cutter	✓				✓	✓	✓		✓
	Prototyping					✓	✓			
	Cutting Machine	✓		✓		✓	✓			
	Project Discuss	✓	✓			✓	✓			✓
	Computer	✓				✓	✓			
	Laser Cutter Teaching	✓				✓	✓			
	Guided Tour	✓	✓	✓						
3D Printer Room	3D Printer Class	✓				✓	✓			
	3D Printer	✓				✓	✓			✓
	CNC Machine	✓		✓						
	Guided Tour	✓		✓						
Teenager Office	Administration	✓	✓							
Teaching Space	lecture		✓		✓		✓			
	Conference	✓	✓		✓					
	Manager Meeting			✓	✓			✓	✓	
	Teaching Assistant Class	✓	✓							✓
Muse Space	Project Discuss					✓				
	Idea Thinking	✓	✓			✓				✓
Kitchen	Cooking	✓			✓		✓			
Woodworking Room	WireSaw Machine	✓				✓	✓	✓	✓	✓
	Sawmill Machine	✓				✓	✓	✓	✓	✓
	Boring Machines			✓				✓		
	Vacuum Forming Machine							✓		
	Planer Machine	✓				✓	✓	✓		✓
	Grinder Machine							✓		
	Lathe Machine			✓		✓		✓	✓	
Scrub Room	Polisher Machine	✓				✓	✓	✓		✓
	Exhaust Fan							✓	✓	
	Sandblasting Machine	✓		✓		✓	✓	✓		✓
	Bandsaw Machine	✓				✓	✓	✓		✓
Metalworking Room	Hand Jointer Machine	✓				✓			✓	✓
	Angle Slot Machine						✓	✓	✓	✓
	Vertical Flower Planer								✓	
Model Room	Polisher Machine	✓		✓						✓
	Hand Jointer Machine	✓		✓		✓		✓		✓
